# Alcohol misuse and outpatient follow-up after hospital discharge: a retrospective cohort study

**DOI:** 10.1186/s13722-018-0125-1

**Published:** 2018-12-04

**Authors:** Bryan Borg, Ivor S. Douglas, Madelyne Hull, Angela Keniston, Marc Moss, Brendan J. Clark

**Affiliations:** 1 Department of Medicine, University of Colorado Anschutz Medical Campus, Aurora, CO USA; 2 Denver Health Medical Center, Denver, CO USA; 3 Division of Pulmonary Sciences and Critical Care Medicine, Department of Medicine, University of Colorado Anschutz Medical Campus, Box C272, RC2, 9th Floor, 12700 East 19th Avenue, Aurora, CO 80045 USA; 4 Division of Substance Dependence, University of Colorado Anschutz Medical Campus, Aurora, CO USA

**Keywords:** Alcohol abuse, Alcoholism, Alcohol use disorder, Alcohol dependence, Hospital readmission, Healthcare utilization, Intensive care unit

## Abstract

**Purpose:**

Patients with alcohol misuse are less likely to receive preventive health services but more likely to utilize emergency health services. However, the association between alcohol misuse and outpatient follow-up after hospitalization is unknown and may depend on whether a patient experiences a critical illness. We sought to determine whether alcohol misuse was associated with lower rates of outpatient follow-up after hospital discharge and whether the magnitude of this association differed in patients who experienced a critical illness.

**Materials and methods:**

This was a retrospective cohort study using administrative data from an urban safety net hospital. Patients were included if they were admitted between 2011 and 2015, were between the ages of 18 and 89, resided within the safety net county, were discharged home, and were at moderate to high risk for hospital readmission within the subsequent 30 days. Alcohol misuse was identified using a combination of ICD-9 codes and response to a single screening question. The primary outcome was a combined measure of follow-up with a primary care physician or specialist provider in the 30 days following hospital discharge. Multivariable logistic regression was used to adjust for factors known to be associated with healthcare utilization.

**Results:**

Overall, 17,575 patients were included in the analysis; 4984 (28%) had alcohol misuse. In the 30 days following hospital discharge, 46% of patients saw any outpatient provider. In an unadjusted analysis, the association between alcohol misuse and attending any outpatient follow-up was dependent on whether patients had a critical illness (p value < 0.0001) with the highest rates of follow-up in survivors of critical illness without alcohol misuse (53%, 95% CI 51%, 55%) followed by patients without alcohol misuse or critical illness (49%; 95% CI 48%, 50%), patients with alcohol misuse without critical illness (38%; 95% CI 36%, 39%), and patients with alcohol misuse and a critical illness (37%; 95% CI 35%, 40%). Adjusting for factors associated with healthcare utilization, these findings were modestly attenuated but unchanged.

**Conclusions:**

Patients with alcohol misuse who are at moderate to high risk for hospital readmission may benefit from targeted interventions to increase rates of outpatient follow-up after hospital discharge.

**Electronic supplementary material:**

The online version of this article (10.1186/s13722-018-0125-1) contains supplementary material, which is available to authorized users.

## Introduction

The spectrum ranging from excessive alcohol use without consequences to the presence of an alcohol use disorder (AUD) is referred to as alcohol misuse [1]. Alcohol misuse predisposes to and can increase the severity of numerous conditions that may require hospitalization including community acquired pneumonia, trauma, gastrointestinal bleeding, sepsis, alcohol withdrawal, and acute respiratory failure [2–5]. Consequently, up to one-third of hospitalized patients have alcohol misuse; most hospitalized patients with alcohol misuse have alcohol misuse [6–12]. Following hospital discharge, patients with alcohol misuse have significantly higher rates of hospital readmission within 30 days [13, 14].

The hospital discharge process is complex and a vulnerable time for patients. Up to 20% of Medicare patients will be readmitted to the hospital within 30 days [15]. Because some of these hospital readmissions are avoidable and add unnecessary cost, there has been an intense effort to reduce rates of hospital readmission. Interventions designed to reduce rates of hospital readmission are often multifaceted [16]. In the pre-discharge time period, these interventions may include patient education, discharge planning, and medication reconciliation [17–19]. Common post-discharge interventions may include a follow-up telephone call, a discharge hotline, and communication with the outpatient provider [17–19]. In addition, some experts argue that multi-faceted interventions would be most effective if they bridged to timely outpatient follow-up [18]. Although there is scant data from randomized controlled trials to support this assertion, several observational studies demonstrate a reduction in hospital readmission for patients who attend follow-up appointments, particularly among patients at high risk for hospital readmission [20–22]. Therefore, identifying groups that are at high risk for not following up with their outpatient providers may identify a group where targeted interventions could improve outcomes.

Prior studies have demonstrated that patients with alcohol misuse over-utilize unplanned emergency services such as emergency department visits and hospital or intensive care unit (ICU) admissions and under-utilize planned, primary care and preventive services such as cancer screening and health maintenance [23–28]. Despite this understanding, there have been no studies focusing on the relationship between alcohol misuse and outpatient follow-up after hospital discharge.

Patterns of healthcare utilization also vary based on whether a patient requires care in an ICU during their hospitalization. Patients may require care in an ICU because of a severe, life-threatening illness that requires additional levels of support or a higher level of monitoring. Following hospital discharge, ICU survivors frequently face a constellation of new or worsening mental and physical health problems including symptoms of anxiety, depression, post-traumatic stress disorder, and loss of physical function [29–33]. Perhaps because of this constellation of new physical and mental health problems, survivors of critical illness have higher rates of healthcare utilization after hospital discharge [34]. It is possible that these higher rates of healthcare utilization in ICU survivors could be attenuated by ongoing alcohol misuse.

Given the high prevalence of alcohol misuse in hospitalized patients, its association with increased morbidity and mortality following hospital discharge, and the known association between outpatient follow-up and improved outcomes, we sought to determine whether alcohol misuse was associated with lower rates of outpatient follow-up after hospital discharge. Furthermore, because survivors of critical illness have higher rates of healthcare utilization, we sought to determine whether the magnitude of this association depended on whether a patient received care in an ICU.

## Materials and methods

### Study design, setting, and data sources

This was a retrospective cohort study which utilized existing data from patients who received care at a Denver Health clinic or at Denver Health Medical Center. Denver Health is a safety net health system that provides care to 25% of all Denver residents, around 150,000 individuals. In the United States, a safety net hospital is one that provides healthcare to patients regardless of their ability to pay. Denver Health also provides training for medical professionals. The population served includes numerous patients who are uninsured, homeless, have psychiatric illness, or drug or alcohol use disorders. Data for admissions were extracted through the Denver Health Data Warehouse, including prescribing, billing, and outcomes data for the 3 years preceding each admission and 30 days following hospital discharge. The Denver Health Data Warehouse contains data for visits at any Denver Health outpatient clinic as well as data from inpatient stays at Denver Health Hospital.

### Study sample

The study sample included patients age ≥ 18 years and ≤ 89 years who were discharged home from Denver Health Hospital after admission from the emergency department or adult urgent care clinic between 1/2011 and 12/2015, resided in Denver County and had a moderate to high risk of hospital readmission within 30 days based on their length of stay in hospital [L], acuity of admission [A], comorbidity [C] and emergency department utilization in the 6 months before admission [E] (LACE + Index). The LACE + Index is a validated model with good discrimination to predict the risk of unplanned readmission or death within 30 days of hospital discharge. A score ≥ 29 suggests at least moderate risk of readmission [35]. A recent review comparing the effectiveness of various 30-readmission risk calculators including the LACE + index found no significant difference in performance between these tools when applied to a large cohort [36]. The LACE + index can be calculated automatically by the electronic records system used at Denver Health. Patients who were admitted to correctional care, psychiatric care, labor and delivery services or were discharged to skilled nursing facilities, long term acute care hospitals, hospice care, or who died in-hospital were excluded. When a patient had multiple admissions during the study period, only the first admission was considered. This study received approval including a waiver of informed consent and Health Insurance Portability and Accountability Act authorization from the Colorado Multiple Institutional Review Board.

### Outcomes

The appropriate venue for follow-up after hospital discharge could include either primary care or specialty care. Therefore, the primary outcome variable was attendance at either within 30 days of hospital discharge [20]. Secondary outcomes included attendance at an outpatient primary care appointment or attendance at an outpatient specialty care appointment considered separately.

### Independent variable

The independent variable of interest was an interaction term between alcohol misuse and critical illness. A priori, we planned to examine the association between alcohol misuse and outpatient follow-up independent of critical illness only if this interaction term was not significant [37]. Patients with International Classification of Diseases (ICD) codes consistent with an alcohol use disorder (ICD-9 prefix 291 or 303, ICD 10 prefix F10.1, F10.2, or F10.9) [38] in the preceding 3 years were considered to have alcohol misuse. In addition, patients who responded yes to the single screening question for alcohol misuse, “In the past 3 months, have you had more than 4 (all women and men > 65) or 5 (Men < 65) alcoholic beverages in 1 day?” were considered to have alcohol misuse. We chose to include screening results in the definition of alcohol misuse because of the limited sensitivity of alcohol codes in administrative data [38]. This question has a sensitivity of 83% and specificity of 72% for past year alcohol use disorder and sensitivity of 86% and specificity of 86% for alcohol misuse [39]. Patients who were admitted to an ICU for any part of their hospital stay were considered to have a critical illness.

### Covariates

Pre-specified co-variates were selected because of their association with health care utilization and included age (continuous), gender, race/ethnicity (White, Black, Hispanic, Other), payer source (Commercial, Self-Pay, Medicare, Medicaid), homelessness and medical co-morbidities measured using the Charlson/Deyo index [40]. As previously described, the Charlson/Deyo index was considered as a categorical variable [41].

### Statistical analysis

Differences between patients with and without alcohol misuse were compared using t tests for normally distributed continuous variables and Chi square tests for categorical variables. To determine whether the association between alcohol misuse and outpatient follow-up was dependent on whether a patient experienced a critical illness, we used the Cochran–Mantel–Haenszel test in unadjusted analyses and an interaction term between alcohol misuse and ICU stay in multivariable analyses. Three separate multivariable logistic regression models were utilized with an interaction between alcohol misuse and critical illness as the predictor variable and any outpatient follow-up, follow-up with a primary care provider, and follow-up with a specialist provider as separate outcomes. Each multivariable analysis was adjusted for the previously outlined pre-specified covariates and included the main effects for alcohol misuse and critical illness. For the analyses using any outpatient follow-up and specialist follow-up as the outcome variable, interaction terms were significant. Therefore, results are displayed by subgroups stratified by alcohol misuse and critical illness. For the analysis using follow-up with a primary care provider as the outcome variable, the interaction term was not significant. Therefore, we present alcohol misuse and critical illness as main effects.

## Results

**Fig. 1:**
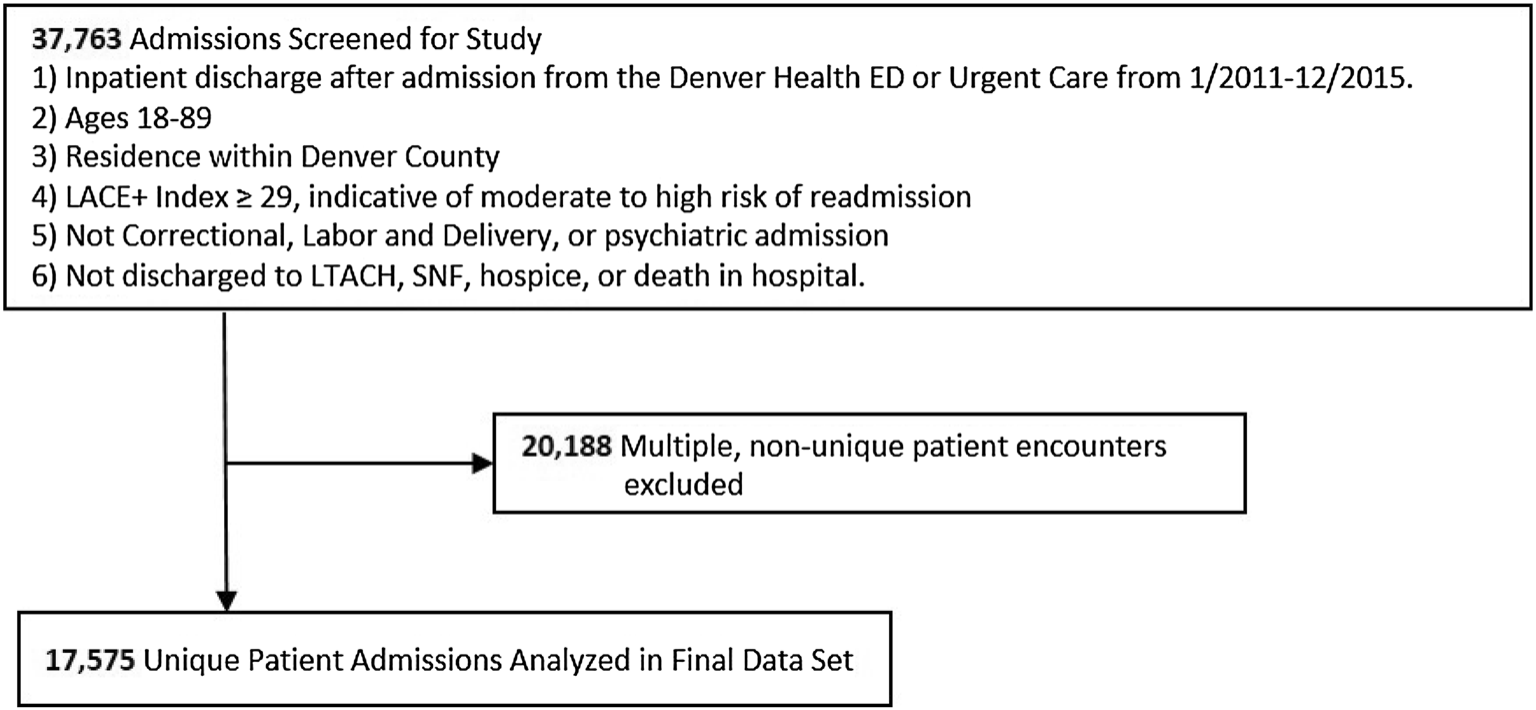
Selection of study sample

Of the 37,763 admissions during the study period who met inclusion criteria, 17,575 were included in this analysis (Fig. 1). Among patients included in the study sample, 28% had alcohol misuse. The average age of the sample was 56.7 years and 58% were male (Table 1). Most patients were white, non-Hispanic (43%) or Hispanic (37%). Almost a quarter of the entire sample was homeless and 16% had a substance use disorder other than alcohol. Patients with alcohol misuse were significantly more likely to be younger, male, homeless, have a concomitant substance use disorder, mental health comorbidities, and liver disease, but lower median Charlson–Deyo Comorbidity score.

**Table 1:** Baseline characteristics

	Overall	Alcohol misuse	No alcohol misuse	p value
	N = 17,575	N = 4984	N = 12,591	
Age (mean)	56.7	53.8	57.9	< 0.0001
Male gender (%)	57.8	68.8	53.5	< 0.0001
Ethnicity				< 0.0001
White (%)	42.7	46.7	41.1	
Black (%)	16.4	15.8	16.6	
Hispanic (%)	36.8	33.3	38.1	
Other (%)	4.2	4.2	4.2	
Homelessness (%)	23.2	34.7	18.7	< 0.0001
Comorbidities				
Other substance use disorder (%)	16.2	24.7	12.5	< 0.0001
Bipolar disorder (%)	9.2	11.4	7.2	< 0.0001
Depression (%)	30.4	33.4	29.1	< 0.0001
Schizophrenia (%)	4.4	5.5	3.9	< 0.0001
Hepatitis C (%)	11.5	17.4	8.9	< 0.0001
HIV/AIDS (%)	2.9	3.7	2.5	< 0.0001
Mild liver disease (no portal hypertension) (%)	5.2	9.9	3.1	< 0.0001
Moderate-severe liver disease (%)	2.9	5.9	1.5	< 0.0001
Payer source				< 0.0001
Self pay/other (%)	26.2	32.3	23.8	
Medicaid (%)	32.5	35.3	31.5	
Medicare (%)	32.9	25.2	36.0	
Commercial (%)	8.3	7.2	8.8	
Charlson/Deyo index				< 0.0001
0 (%)	5.6	8.7	4.3	
1–2 (%)	45.7	51.0	43.6	
≥ 3 (%)	48.7	40.3	52.1	
Length of stay (days)	4.1	4.4	4.0	< 0.01

*AIDS* acquired immunodeficiency syndrome, *HIV* human immunodeficiency virus, *Charlson/Deyo index* a measure of medical comorbidities

In the 30 days following hospital discharge, 46% of patients saw any outpatient provider, 34% saw a primary care provider, 23% saw a specialty provider, and 11% saw both types of providers. Males, patients who were homeless, and patients with substance use and mental health disorders had significantly lower rates of outpatient follow-up. In unadjusted analyses, the associations between alcohol misuse and attending any outpatient follow-up as well follow-up with a primary care provider or a specialist were all dependent on whether patients had a critical illness (Cochran–Mantel–Haenszel p value < 0.0001). Patients with alcohol misuse who experienced a critical illness had the lowest rates of any outpatient follow-up and primary care physician follow-up (Table 2). In contrast, survivors of critical illness without alcohol misuse had significantly higher rates of outpatient follow-up when compared to patients without alcohol misuse who did not have a critical illness (p < 0.001). The high rates of outpatient follow-up for this group were driven by both higher rates of follow-up with a primary care provider and a specialist.

**Table 2:** Unadjusted rates of outpatient follow-up within 30 days of hospital discharge

	Any outpatient provider*		Primary care physician*		Specialist physician*	
	%, 95% CI	p value	%, 95% CI	p value	%, 95% CI	p value
Critical illness						
No alcohol misuse (n = 2840)	53 (51, 55)	Ref	31 (29, 33)	Ref	35 (34, 37)	Ref
Alcohol misuse (n = 1263)	37 (35, 40)	< 0.001	24 (22, 27)	< 0.001	22 (20, 24)	< 0.001
No critical illness						
No alcohol misuse (n = 9751)	49 (48, 50)	Ref	37 (36, 38)	Ref	23 (22, 23)	Ref
Alcohol misuse (n = 3721)	38 (36, 39)	< 0.001	29 (27, 30)	< 0.001	16 (15, 17)	< 0.001

**Cochran–Mantel–Haenszel p value < 0.0001 when stratifying relationship between alcohol misuse and outcome by critical illness

Adjusted for age, gender, race/ethnicity, payer source, medical comorbidities, and homelessness, the association between alcohol misuse and any outpatient follow-up remained dependent on whether a patient had been admitted to an ICU during their hospital stay (p value for interaction term < 0.01). Among the 4103 patients admitted to an ICU, the odds of outpatient follow-up were significantly lower in patients with alcohol misuse when compared to those without (OR 0.60; 95% CI 0.52, 0.70) (Table 3). Among the 13,472 patients who were not admitted to an ICU during their hospital stay, patients with alcohol misuse were also less likely to attend any outpatient follow-up though the association was not as strong (OR 0.76; 95% CI 0.70, 0.83).

**Table 3:** Unadjusted and adjusted association between alcohol misuse and any outpatient follow-up as well as follow-up with a specialist provider in the 30 days following hospital discharge among patients who were admitted to an intensive care unit during their hospital stay

	Any outpatient follow-up (n = 4103)		Specialist provider (n = 4103)	
	Unadjusted OR (95% CI)	Adjusted OR (95% CI)	Unadjusted OR (95% CI)	Adjusted OR (95% CI)
Alcohol misuse	0.54 (0.47, 0.61)^Z^	0.60 (0.52, 0.70)^Z^	0.66 (0.60, 0.73)^Z^	0.55 (0.47, 0.64)^Z^
Age		1.01 (1.00, 1.02)**		1.00 (1.00, 1.01)
Gender (male)		0.81 (0.71, 0.93)**		1.18 (1.02, 1.37)**
Race				
White, non-hisp		Ref		Ref
Black		1.33 (1.0, 1.61)**		1.05 (0.85, 1.30)
Hispanic		1.79 (1.55, 2.07)^Z^		1.29 (1.11, 1.50)*
Other		1.29 (0.93, 1.78)		1.36 (0.97, 1.90)
Payer source				
Commercial		Ref		Ref
Medicaid		1.27 (1.02, 1.59)**		0.82 (0.65, 1.03)
Medicare		0.75 (0.60, 0.96)**		0.54 (0.42, 0.69)^z^
Self-pay/other		0.97 (0.77, 1.22)		0.78 (0.62, 0.99)**
Homeless		0.56 (0.48, 0.66)^Z^		0.53 (0.44, 0.64)^Z^
Charlson/Deyo				
0		Ref		Ref
1–2		1.02 (0.79, 1.30)		0.79 (0.61, 1.02)
≥ 3		1.26 (0.96, 1.65)		1.05 (0.79, 1.40)

Charlson/Deyo index is a measure of medical comorbidities

*OR* odds ratio

^z^p < 0.0001; *< 0.001; **< 0.05

Similarly, after adjusting for age, gender, race/ethnicity, payer source, medical comorbidities, and homelessness, the association between alcohol misuse and specialist follow-up remained dependent on whether patients had been admitted to an ICU (p value for interaction = 0.004). Among patients admitted to an ICU during their hospital stay, the odds of follow-up with a specialist provider were significantly lower in patients with alcohol misuse when compared to those without (OR 0.55; 95% CI 0.47, 0.64) (Table 3). Among the patients who were not admitted to an ICU during their hospital stay, patients with alcohol misuse had significantly lower odds of outpatient follow-up with a specialist provider (OR 0.69; 95% CI 0.62, 0.76) (Table 4).

**Table 4:** Unadjusted and adjusted association between alcohol misuse and any outpatient follow-up as well as follow-up with a specialist provider in the 30 days following hospital discharge among patients who were not admitted to an intensive care unit during their hospital stay

	Any outpatient follow-up (n = 13,472)		Specialist provider (n = 13,472)	
	Unadjusted OR (95% CI)	Adjusted OR (95% CI)	Unadjusted OR (95% CI)	Adjusted OR (95% CI)
Alcohol misuse	0.64 (0.59, 0.69)^Z^	0.76 (0.70, 0.83)^Z^	0.66 (0.60, 0.73)^Z^	0.69 (0.62, 0.76)^Z^
Age		1.00 (0.99, 1.00)		0.98 (0.98, 0.99)^Z^
Gender (male)		0.67 (0.63, 0.72)^Z^		0.95 (0.87, 1.03)
Race				
White, non-Hispanic		Ref		Ref
Black		1.61 (1.46, 1.79)^Z^		1.19 (1.06, 1.35)**
Hispanic		1.97 (1.82, 2.14)^Z^		1.1.33(1.21, 1.47)^Z^
Other		1.10 (0.91, 1.31)		1.12 (0.90, 1.40)
Payer source				
Commercial		Ref		Ref
Medicaid		1.85 (1.59, 2.16)^Z^		1.19 (0.99, 1.42)
Medicare		1.53 (1.31, 1.78)^Z^		0.97 (0.80, 1.17)
Self-pay/other		1.25 (1.07, 1.46)^Z^		1.02 (0.85, 1.23)
Homeless		0.62 (0.57, 0.68)^Z^		0.68 (0.61, 0.76)^Z^
Charlson/Deyo				
0		Ref		Ref
1–2		0.99 (0.83, 1.19)		0.74 (0.61, 0.91)**
≥ 3		1.40 (1.16, 1.70)*		1.13 (0.79, 1.40)

Charlson/Deyo index is a measure of medical comorbidities

*OR* odds ratio

^z^p < 0.0001; *< 0.001; **< 0.05

In a fully adjusted analysis, the relationship between alcohol misuse and follow-up with a primary care physician was not dependent on whether a patient experienced a critical illness. However, patients with alcohol misuse (OR 0.85; 95% CI 0.79, 0.92) and survivors of critical illness (OR 0.87; 95% CI 0.81, 0.95) were significantly less likely to see a primary care physician (Table 5).

**Table 5:** The relationship between alcohol misuse and follow-up with a primary care physician did not depend on whether a patient had a critical illness

	Primary care physician	
	Unadjusted OR (95% CI)	Adjusted OR (95% CI)
No alcohol misuse	Ref	Ref
Alcohol misuse	0.69 (0.64, 0.74)^Z^	0.85 (0.79, 0.92)^Z^
No critical illness	Ref	Ref
Critical illness	0.77 (0.71, 0.83)^Z^	0.87 (0.81, 0.95)^Z^
Age		1.01 (1.00, 1.01)^Z^
Gender (male)		0.63 (0.0.59, 0.68)^Z^
Race		
White, non-hisp		Ref
Black		1.65 (1.50, 1.82)^Z^
Hispanic		2.07 (1.92, 2.23)^Z^
Other		1.11 (0.94, 1.32)^a^
Payer source		
Commercial		Ref
Medicaid		2.18 (1.89, 2.52)^Z^
Medicare		1.84 (1.59, 2.13)^Z^
Self-pay/other		1.28 (1.10, 1.49)
Homeless		0.72 (0.66, 0.78)^Z^
Charlson/Deyo		
0		Ref
1–2		1.53 (1.28, 1.83)
≥ 3		1.93 (1.60, 2.34)^Z^

Charlson/Deyo index is a measure of medical comorbidities

*OR* odds ratio

^z^p < 0.0001; *< 0.001; **< 0.05

A key assumption in this analysis was that access to care was equal across groups. To test this assumption, we conducted a sensitivity analysis in the subset of patients who had seen a primary care provider within the system in the 3 years preceding the index admission. The inferences were unchanged. The adjusted odds of outpatient follow-up with any provider and the odds of follow-up with a primary care provider were lower than those seen in the overall cohort (Additional file 1: Table S1).

## Discussion

In this retrospective cohort study of over 17,000 patients who received care in a single metropolitan health network, we sought to determine whether patients with alcohol misuse were less likely to attend outpatient follow-up visits after hospitalization for a medical illness. We found that, overall, less than half of patients at moderate to high risk of hospital readmission saw any outpatient provider in the 30 days after hospital discharge. However, patients who experienced a critical illness without alcohol misuse had significantly higher rates of follow-up. Conversely, patients with alcohol misuse consistently had lower rates of outpatient follow-up regardless of whether they experienced a critical illness. It is possible that we did not observe higher rates of follow-up in ICU survivors with alcohol misuse because they do not differentially decrease their alcohol consumption when compared to patients with alcohol misuse who were not admitted to an ICU.

These findings have several potential limitations. First, by defining alcohol misuse using ICD-9 codes or a positive response to a single screening question, we may be combining patients with at-risk alcohol use and those with an alcohol use disorder. Prior work in a similar setting (an urban safety net hospital) demonstrated that 77% of medical inpatients enrolled in a study of brief intervention for alcohol misuse had DSM-IV alcohol dependence [42]. Therefore, even with the limitations of our definition of alcohol misuse, it is very likely that many patients that we identified as having alcohol misuse had an AUD. Unfortunately, within the patients we identify as having alcohol misuse, there is no reliable way to determine which had at-risk use and which had an AUD.

Second, it is probable that rates of outpatient follow-up were underestimated since we were only able to account for follow-up within the Denver Health system. However, it is unlikely that this occurred differentially between patients with and without alcohol misuse. Third, we did not account for post-discharge out-of-hospital death in our analysis. Death is a competing risk for outpatient follow-up. However, patients with alcohol misuse have a higher risk of death following hospital discharge so it is likely that this would lead to an underestimation of the true effect size [43]. Fourth, although we based our primary inference on a statistical analysis that accounted for differences in socioeconomic status and our findings persisted in a subgroup of patients with known access to outpatient care, there is the possibility that our findings are due to residual or unmeasured confounding. Fifth, because this study used administrative data, it is possible that some follow-up visits were unrelated to the hospitalization and, thus, do not reflect a continuum of care. Furthermore, with the available data, we were unable to distinguish whether an appointment was made and not kept or never made at all. Finally, this study was conducted using a sample that receives care at a safety net hospital. Patients in this sample are generally of lower socioeconomic status. Therefore, these findings may not apply to other hospital systems.

The lower rates of outpatient follow-up after hospital discharge among patients with alcohol misuse are consistent with prior studies demonstrating that patients with alcohol misuse are less likely to receive preventive health care [44]. One potential explanation for the lower rates of follow-up among patients with alcohol misuse could be that patients who are actively intoxicated are less likely to receive care. Alternatively, patients with alcohol misuse may actively avoid interactions with healthcare providers because of shame, because they feel embarrassed, because they did not want to engage in discussions about their drinking, or because drinking may have a higher priority in their life [45].

Finally, it is possible that patients and/or providers perceive that there is no need for outpatient follow-up. For example, patients and providers may perceive that an illness is solely related to alcohol, agree that the plan should be to cut down or stop drinking, and, therefore, decide that no additional follow-up is indicated. This may lead to a missed opportunity for patients with alcohol misuse for several reasons. Given the high rate of AUDs among these hospitalized patients, there may be the opportunity to effectively manage AUDs in the primary care setting. Though data on this is conflicting, some clinical trials demonstrate effectiveness [46–48]. For patients with at-risk use, follow-up with a primary care physician would offer an opportunity re-evaluate drinking, thus delivering a multi-contact intervention. These brief multi-contact interventions have the strongest evidence base [49]. Second, many patients who are hospitalized for an illness may need chronic medications to manage their condition. Examples of this include patients with congestive heart failure or chronic obstructive pulmonary disease. Given prior data that demonstrates higher rates of medication non-adherence among patients with alcohol misuse, outpatient follow-up may provide an opportunity to identify and overcome barriers to adherence [50].

Our findings suggest that it may be important to tailor interventions to increase outpatient follow-up following hospital discharge for patients with alcohol misuse. Previously described interventions to increase rates of outpatient follow-up after hospital discharge do not account for potentially unique barriers such as shame, embarrassment, stigma, difficulties building therapeutic alliance, and psychiatric comorbidities that may prevent patients with alcohol misuse from following up with outpatient providers [51–53]. Tailoring a care transition intervention to patients with alcohol misuse could lead to improved outcomes at a lower cost, thus improving the value of care. While the optimal method for linking patients with alcohol misuse to care outside of the inpatient setting is unclear, promising approaches include the use of incentives, introducing the patient to the clinician who they will see after hospital discharge, and providing an escort to the patient’s first appointment [54–57].

The finding that survivors of critical illness with alcohol misuse have the lowest rates of outpatient follow-up is concerning. When compared to survivors of critical illness without alcohol misuse, survivors of critical illness with alcohol misuse have a higher risk of morbidity and mortality following hospital discharge [14]. This higher risk of morbidity and mortality is likely driven by a combination of new or worsening chronic health problems and underlying mental health problems [58]. Given the higher risk of poor outcomes in this population, rates of outpatient follow-up among survivors of critical illness with alcohol misuse should be comparable or higher than those without alcohol misuse.

## Conclusions

This study demonstrates that patients with alcohol misuse who are at moderate to high risk for hospital readmission have lower rates of follow-up within 30 days of hospital discharge. Rates of follow-up for survivors of critical illness with alcohol misuse, a group known to be at higher risk for morbidity and mortality, were disappointingly low. Ongoing efforts to improve the bridge to outpatient longitudinal care for patients with alcohol misuse could reduce readmissions, decrease healthcare costs, and improve outcomes.

## Additional file

**Additional file 1: Table S1.** Unadjusted and multivariable analyses within population that had seen any Primary Care clinic in preceding 3 years examining the association between alcohol misuse and any outpatient follow-up, follow-up with a primary care provider, and follow-up with a specialist.

## Abbreviations

AUD: alcohol use disorder
ICU: intensive care unit
LACE: length of stay, acuity comorbidity, emergency department utilization
HIPAA: Health Insurance Portability and Accountability Act
ICD: International Classification of Diseases
