# Supplementary material for: Alcohol misuse and outpatient follow-up after hospital discharge: a retrospective cohort study
Source: Addict Sci Clin Pract. 2018 Dec 4;13:24. doi: 10.1186/s13722-018-0125-1 (PMC6278064; doi:10.1186/s13722-018-0125-1)
Supplement: Supplementary file 1 — Additional file 1: Table S1. Unadjusted and multivariable analyses within population that had seen any Primary Care clinic in preceding 3 years examining the association between alcohol misuse and any outpatient follow-up, follow-up with a primary care provider, and follow-up with a specialist. [file 13722_2018_125_MOESM1_ESM.docx]

| **Table S1.** Unadjusted and multivariable analyses **within population that had seen any Primary Care clinic in preceding 3 years** examining the association between alcohol misuse and any outpatient follow-up, follow-up with a primary care provider, and follow-up with a specialist. | | | | | | |
| --- | --- | --- | --- | --- | --- | --- |
|  | **Any Outpatient Follow-up** | | **Primary Care Provider** | | **Specialist Provider** | |
| **N=7,946** | **Unadjusted OR**  **(95% CI)** | **Adjusted OR**  **(95% CI)** | **Unadjusted OR**  **(95% CI)** | **Adjusted OR**  **(95% CI)** | **Unadjusted OR**  **(95% CI)** | **Adjusted OR**  **(95% CI)** |
| **Alcohol misuse** |  |  |  |  |  |  |
| Floor, no alcohol misuse | Ref | Ref | Ref | Ref | Ref | Ref |
| Floor, alcohol misuse | 0.52 (0.47, 0.59) **^Z^** | 0.61 (0.54, 0.69)**^Z^** | 0.61 (0.55, 0.69) **^Z^** | 0.71 (0.63, 0.80) **^Z^** | 0.69 (0.60, 0.78) **^Z^** | 0.70 (0.61, 0.80) **^Z^** |
| ICU, no alcohol misuse | 1.19 (1.02, 1.41)***** | 1.26 (1.07, 1.49)****** | 0.96 (0.83, 1.10) | 1.03 (0.89, 1.19) | 1.72 (1.50, 1.97) **^Z^** | 1.63 (1.42, 1.88) **^Z^** |
| ICU, alcohol misuse | 0.52 (0.42, 0.64) **^Z^** | 0.64 (0.51, 0.79) **^Z^** | 0.56 (0.46, 0.68) **^Z^** | 0.70 (0.57, 0.86) **^Z^** | 0.94 (0.76, 1.17) | 0.91 (0.72, 1.13) |
| **Age** |  | 1.01 (1.01, 1.02) **^Z^** |  | 1.02 (1.01, 1.02) **^Z^** |  | 0.99 (0.98, 0.99) **^Z^** |
| **Gender (Male)** |  | 0.78 (0.71, 0.87) **^Z^** |  | 0.77 (0.70, 0.85) **^Z^** |  | 1.12 (1.01, 1.24)***** |
| **Race** |  |  |  |  |  |  |
| White, non-hisp |  | Ref |  | Ref |  | Ref |
| Black |  | 0.92 (0.79, 1.06) |  | 0.90 (0.79, 1.03) |  | 0.94 (0.82, 1.09) |
| Hispanic |  | 1.15 (1.02, 1.29)***** |  | 1.14 (1.02, 1.27)***** |  | 1.04 (0.93, 1.17) |
| Other |  | 0.90 (0.69, 1.20) |  | 0.90 (0.69, 1.17) |  | 1.13 (0.86, 1.48) |
| **Payer source** |  |  |  |  |  |  |
| Commercial |  | Ref |  | Ref |  | Ref |
| Medicaid |  | 0.99 (0.76, 1.28) |  | 1.17 (0.92, 1.47) |  | 0.66 (0.52, 0.83) **^Z^** |
| Medicare |  | 0.90 (0.69, 1.17) |  | 1.03 (0.81, 1.31) |  | 0.64 (0.51, 0.81) **^Z^** |
| Self-Pay/Other |  | 0.80 (0.61, 1.04) |  | 0.87 (0.69, 1.11) |  | 0.61 (0.47, 0.78) **^Z^** |
| **Homeless** |  | 0.74 (0.65, 0.85) **^Z^** |  | 0.91 (0.80, 1.03) |  | 0.74 (0.64, 0.86) **^Z^** |
| CCI 0 |  | Ref |  | Ref |  | Ref |
| CCI 1-2 |  | 1.01 (0.77, 1.32) |  | 1.40 (1.08, 1.81) ***** |  | 0.72 (0.55, 0.95) ***** |
| CCI≥3 |  | 1.21 (0.91, 1.61) |  | 1.55 (1.18, 2.03) ****** |  | 1.01 (0.76, 1.34) |

*****p<0.05, ******p<0.01, **^Z^**p<0.001
